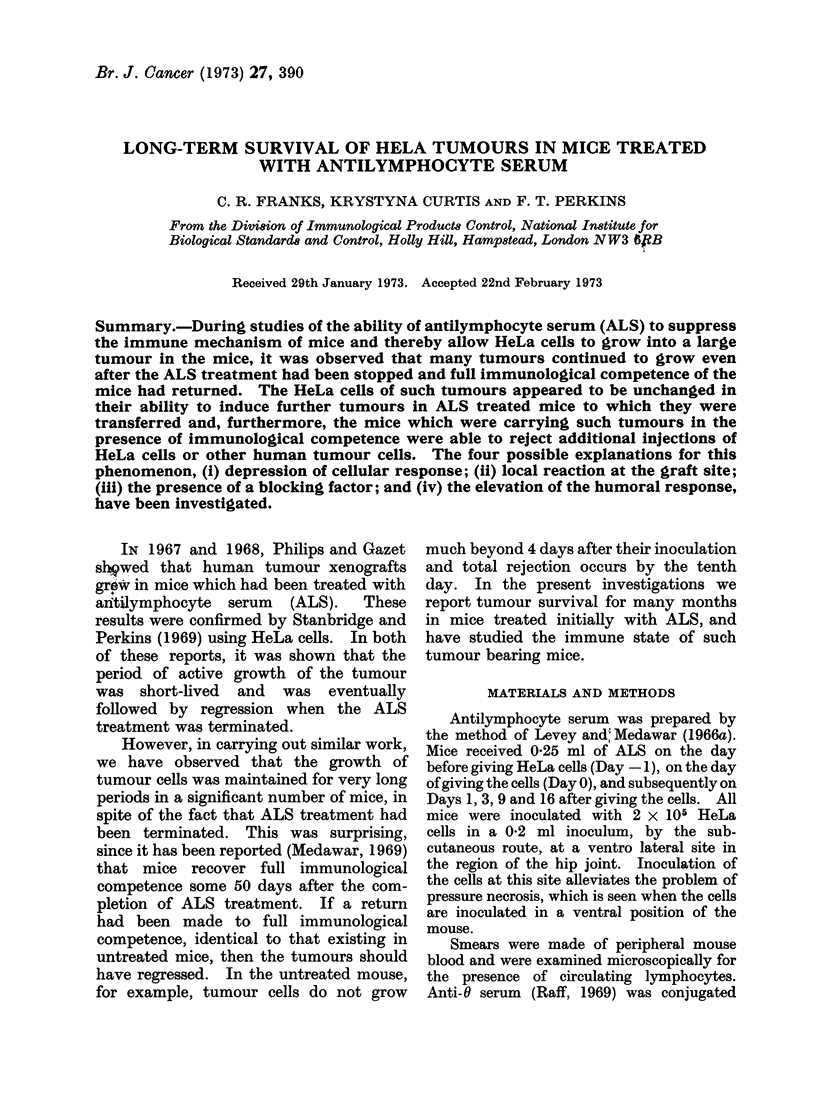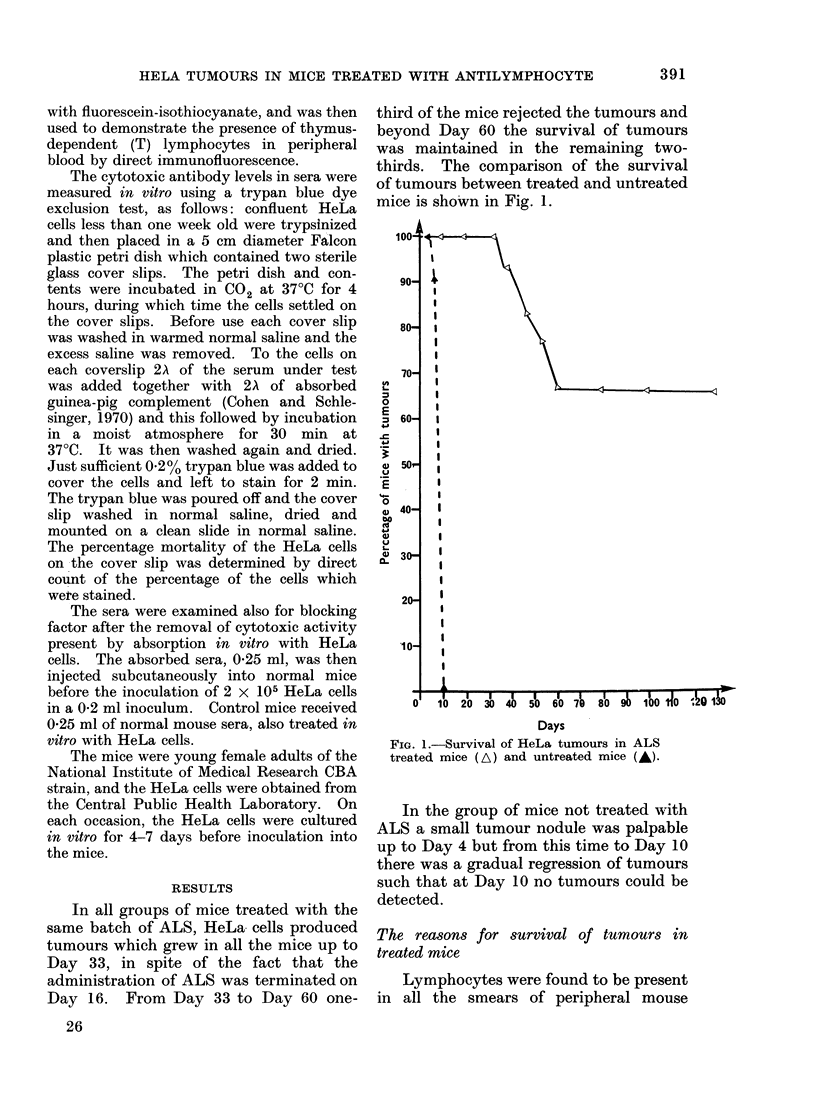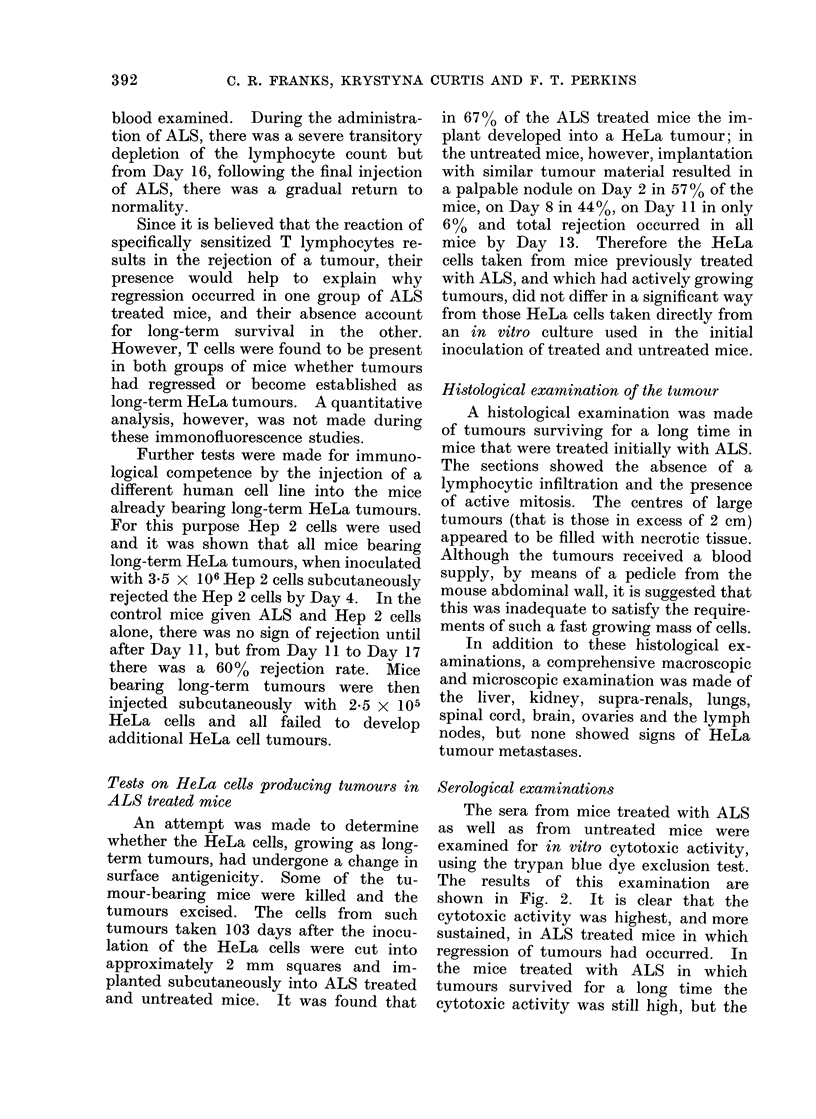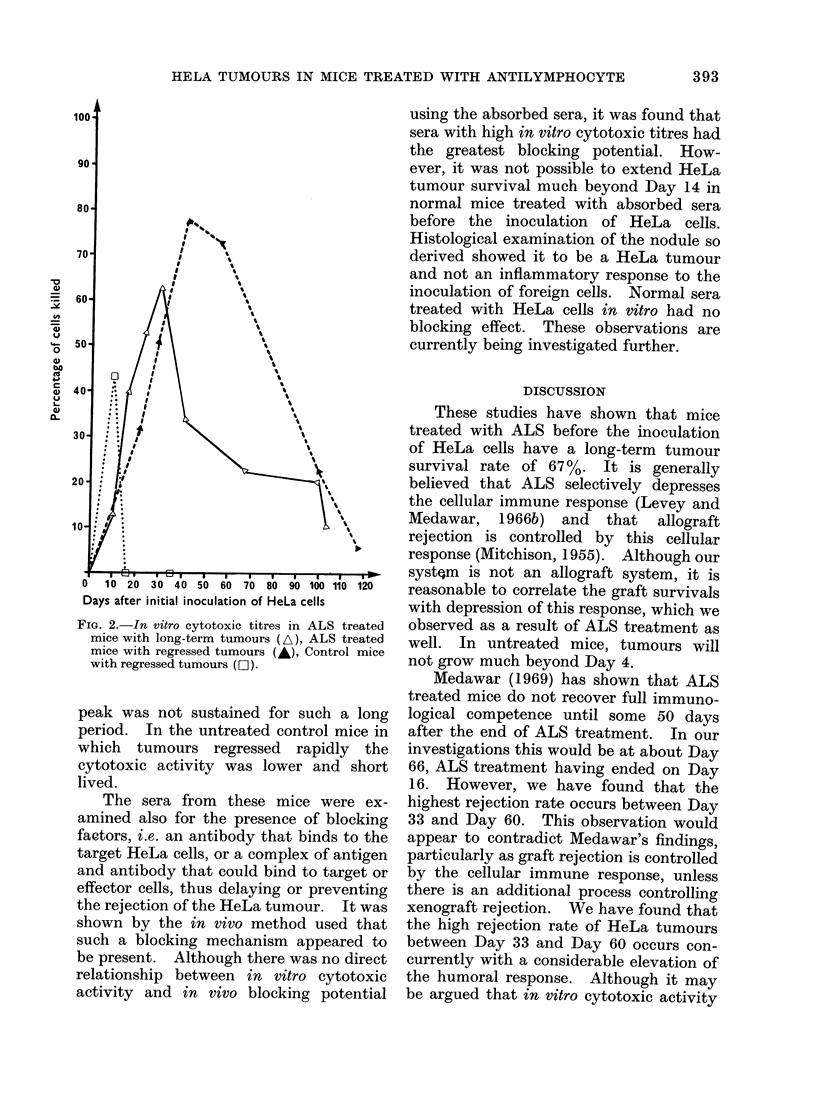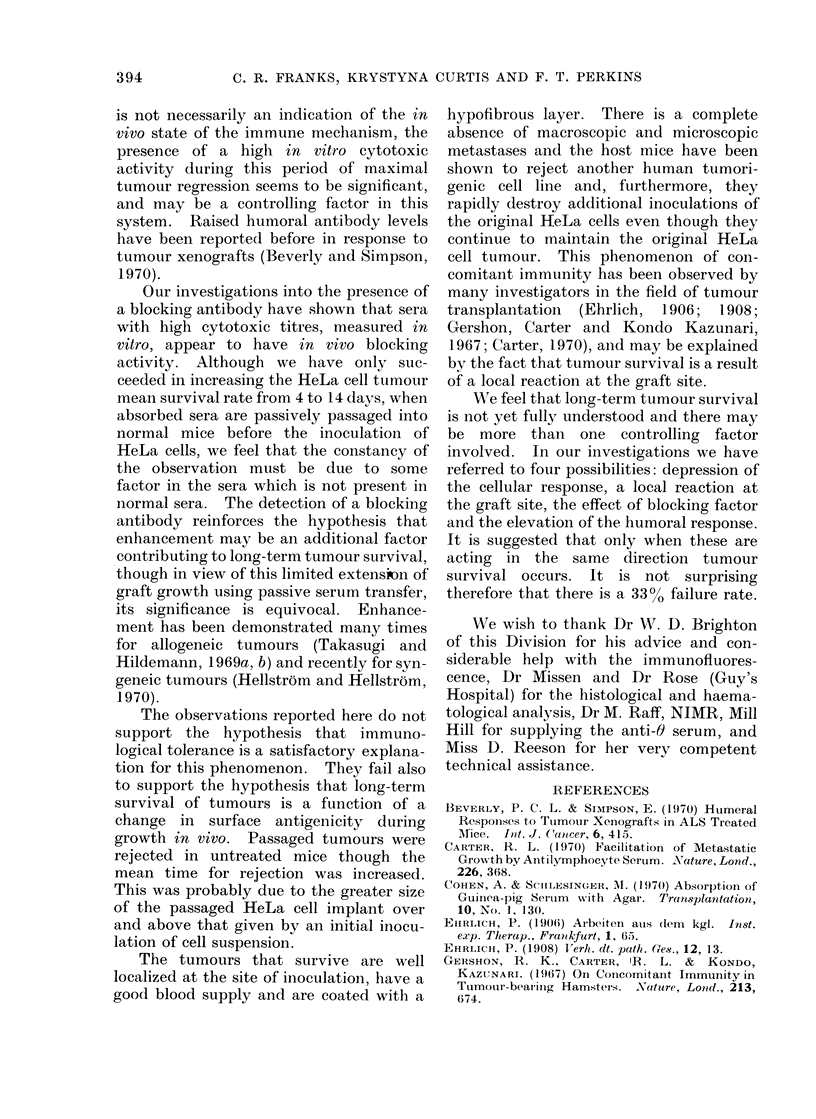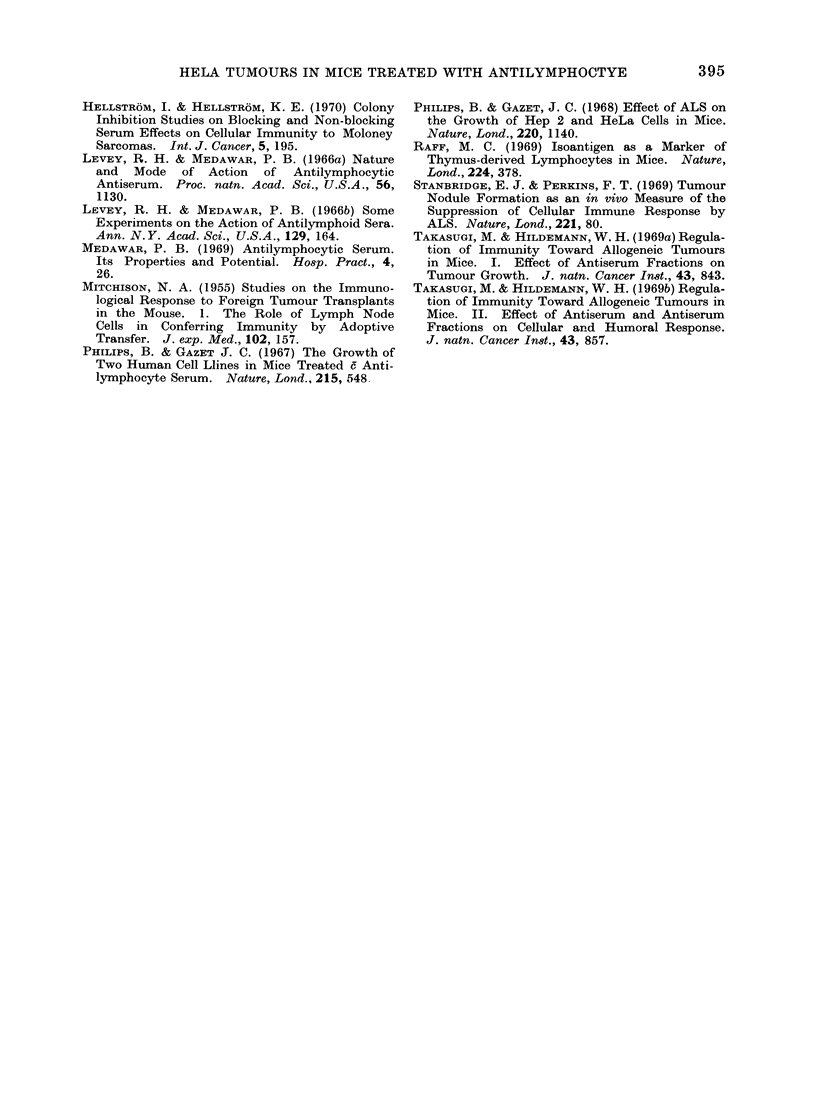# Long-term Survival of HeLa Tumours in Mice Treated with Antilymphocyte Serum

**DOI:** 10.1038/bjc.1973.46

**Published:** 1973-05

**Authors:** C. R. Franks, Krystyna Curtis, F. T. Perkins

## Abstract

During studies of the ability of antilymphocyte serum (ALS) to suppress the immune mechanism of mice and thereby allow HeLa cells to grow into a large tumour in the mice, it was observed that many tumours continued to grow even after the ALS treatment had been stopped and full immunological competence of the mice had returned. The HeLa cells of such tumours appeared to be unchanged in their ability to induce further tumours in ALS treated mice to which they were transferred and, furthermore, the mice which were carrying such tumours in the presence of immunological competence were able to reject additional injections of HeLa cells or other human tumour cells. The four possible explanations for this phenomenon, (i) depression of cellular response; (ii) local reaction at the graft site; (iii) the presence of a blocking factor; and (iv) the elevation of the humoral response, have been investigated.


Br. J. Cancer (1973) 27, 390

### LONG-TERM SURVIVAL OF HELA TUMOURS IN MICE TREATED WITH ANTILYMPHOCYTE SERUM

C. R. FRANKS, KRYSTYNA CURTIS AND F. T. PERKINS

*From the Division of Immunological Products Control, National Institute for Biological Standards and Control, Holly Hill, Hampstead, London NW3 6RB*

Received 29th January 1973. Accepted 22nd February 1973

**Summary.—During studies of the ability of antilymphocyte serum (ALS) to suppress the immune mechanism of mice and thereby allow HeLa cells to grow into a large tumour in the mice, it was observed that many tumours continued to grow even after the ALS treatment had been stopped and full immunological competence of the mice had returned. The HeLa cells of such tumours appeared to be unchanged in their ability to induce further tumours in ALS treated mice to which they were transferred and, furthermore, the mice which were carrying such tumours in the presence of immunological competence were able to reject additional injections of HeLa cells or other human tumour cells. The four possible explanations for this phenomenon, (i) depression of cellular response; (ii) local reaction at the graft site; (iii) the presence of a blocking factor; and (iv) the elevation of the humoral response, have been investigated.**

IN 1967 and 1968, Philips and Gazet showed that human tumour xenografts grew in mice which had been treated with antilymphocyte serum (ALS). These results were confirmed by Stanbridge and Perkins (1969) using HeLa cells. In both of these reports, it was shown that the period of active growth of the tumour was short-lived and was eventually followed by regression when the ALS treatment was terminated.

However, in carrying out similar work, we have observed that the growth of tumour cells was maintained for very long periods in a significant number of mice, in spite of the fact that ALS treatment had been terminated. This was surprising, since it has been reported (Medawar, 1969) that mice recover full immunological competence some 50 days after the completion of ALS treatment. If a return had been made to full immunological competence, identical to that existing in untreated mice, then the tumours should have regressed. In the untreated mouse, for example, tumour cells do not grow much beyond 4 days after their inoculation and total rejection occurs by the tenth day. In the present investigations we report tumour survival for many months in mice treated initially with ALS, and have studied the immune state of such tumour bearing mice.

### MATERIALS AND METHODS

Antilymphocyte serum was prepared by the method of Levey and Medawar (1966*a*). Mice received 0·25 ml of ALS on the day before giving HeLa cells (Day −1), on the day of giving the cells (Day 0), and subsequently on Days 1, 3, 9 and 16 after giving the cells. All mice were inoculated with $2 \times 10^5$ HeLa cells in a 0·2 ml inoculum, by the subcutaneous route, at a ventro lateral site in the region of the hip joint. Inoculation of the cells at this site alleviates the problem of pressure necrosis, which is seen when the cells are inoculated in a ventral position of the mouse.

Smears were made of peripheral mouse blood and were examined microscopically for the presence of circulating lymphocytes. Anti-$\theta$ serum (Raff, 1969) was conjugated with fluorescein-isothiocyanate, and was then used to demonstrate the presence of thymus-dependent (T) lymphocytes in peripheral blood by direct immunofluorescence.

The cytotoxic antibody levels in sera were measured *in vitro* using a trypan blue dye exclusion test, as follows: confluent HeLa cells less than one week old were trypsinized and then placed in a 5 cm diameter Falcon plastic petri dish which contained two sterile glass cover slips. The petri dish and contents were incubated in $CO_2$ at 37°C for 4 hours, during which time the cells settled on the cover slips. Before use each cover slip was washed in warmed normal saline and the excess saline was removed. To the cells on each coverslip 2λ of the serum under test was added together with 2λ of absorbed guinea-pig complement (Cohen and Schlesinger, 1970) and this followed by incubation in a moist atmosphere for 30 min at 37°C. It was then washed again and dried. Just sufficient 0·2% trypan blue was added to cover the cells and left to stain for 2 min. The trypan blue was poured off and the cover slip washed in normal saline, dried and mounted on a clean slide in normal saline. The percentage mortality of the HeLa cells on the cover slip was determined by direct count of the percentage of the cells which were stained.

The sera were examined also for blocking factor after the removal of cytotoxic activity present by absorption *in vitro* with HeLa cells. The absorbed sera, 0·25 ml, was then injected subcutaneously into normal mice before the inoculation of $2 \times 10^5$ HeLa cells in a 0·2 ml inoculum. Control mice received 0·25 ml of normal mouse sera, also treated *in vitro* with HeLa cells.

The mice were young female adults of the National Institute of Medical Research CBA strain, and the HeLa cells were obtained from the Central Public Health Laboratory. On each occasion, the HeLa cells were cultured *in vitro* for 4–7 days before inoculation into the mice.

### RESULTS

In all groups of mice treated with the same batch of ALS, HeLa cells produced tumours which grew in all the mice up to Day 33, in spite of the fact that the administration of ALS was terminated on Day 16. From Day 33 to Day 60 one-third of the mice rejected the tumours and beyond Day 60 the survival of tumours was maintained in the remaining two-thirds. The comparison of the survival of tumours between treated and untreated mice is shown in Fig. 1.

FIG. 1.—Survival of HeLa tumours in ALS treated mice (△) and untreated mice (▲).

In the group of mice not treated with ALS a small tumour nodule was palpable up to Day 4 but from this time to Day 10 there was a gradual regression of tumours such that at Day 10 no tumours could be detected.

*The reasons for survival of tumours in treated mice*

Lymphocytes were found to be present in all the smears of peripheral mouse blood examined. During the administration of ALS, there was a severe transitory depletion of the lymphocyte count but from Day 16, following the final injection of ALS, there was a gradual return to normality.

Since it is believed that the reaction of specifically sensitized T lymphocytes results in the rejection of a tumour, their presence would help to explain why regression occurred in one group of ALS treated mice, and their absence account for long-term survival in the other. However, T cells were found to be present in both groups of mice whether tumours had regressed or become established as long-term HeLa tumours. A quantitative analysis, however, was not made during these immonofluorescence studies.

Further tests were made for immunological competence by the injection of a different human cell line into the mice already bearing long-term HeLa tumours. For this purpose Hep 2 cells were used and it was shown that all mice bearing long-term HeLa tumours, when inoculated with $3{\cdot}5 \times 10^6$ Hep 2 cells subcutaneously rejected the Hep 2 cells by Day 4. In the control mice given ALS and Hep 2 cells alone, there was no sign of rejection until after Day 11, but from Day 11 to Day 17 there was a 60% rejection rate. Mice bearing long-term tumours were then injected subcutaneously with $2{\cdot}5 \times 10^5$ HeLa cells and all failed to develop additional HeLa cell tumours.

### *Tests on HeLa cells producing tumours in ALS treated mice*

An attempt was made to determine whether the HeLa cells, growing as long-term tumours, had undergone a change in surface antigenicity. Some of the tumour-bearing mice were killed and the tumours excised. The cells from such tumours taken 103 days after the inoculation of the HeLa cells were cut into approximately 2 mm squares and implanted subcutaneously into ALS treated and untreated mice. It was found that in 67% of the ALS treated mice the implant developed into a HeLa tumour; in the untreated mice, however, implantation with similar tumour material resulted in a palpable nodule on Day 2 in 57% of the mice, on Day 8 in 44%, on Day 11 in only 6% and total rejection occurred in all mice by Day 13. Therefore the HeLa cells taken from mice previously treated with ALS, and which had actively growing tumours, did not differ in a significant way from those HeLa cells taken directly from an *in vitro* culture used in the initial inoculation of treated and untreated mice.

### *Histological examination of the tumour*

A histological examination was made of tumours surviving for a long time in mice that were treated initially with ALS. The sections showed the absence of a lymphocytic infiltration and the presence of active mitosis. The centres of large tumours (that is those in excess of 2 cm) appeared to be filled with necrotic tissue. Although the tumours received a blood supply, by means of a pedicle from the mouse abdominal wall, it is suggested that this was inadequate to satisfy the requirements of such a fast growing mass of cells.

In addition to these histological examinations, a comprehensive macroscopic and microscopic examination was made of the liver, kidney, supra-renals, lungs, spinal cord, brain, ovaries and the lymph nodes, but none showed signs of HeLa tumour metastases.

### *Serological examinations*

The sera from mice treated with ALS as well as from untreated mice were examined for *in vitro* cytotoxic activity, using the trypan blue dye exclusion test. The results of this examination are shown in Fig. 2. It is clear that the cytotoxic activity was highest, and more sustained, in ALS treated mice in which regression of tumours had occurred. In the mice treated with ALS in which tumours survived for a long time the cytotoxic activity was still high, but the

FIG. 2.—*In vitro* cytotoxic titres in ALS treated mice with long-term tumours (△), ALS treated mice with regressed tumours (▲), Control mice with regressed tumours (□).

peak was not sustained for such a long period. In the untreated control mice in which tumours regressed rapidly the cytotoxic activity was lower and short lived.

The sera from these mice were examined also for the presence of blocking factors, *i.e.* an antibody that binds to the target HeLa cells, or a complex of antigen and antibody that could bind to target or effector cells, thus delaying or preventing the rejection of the HeLa tumour. It was shown by the *in vivo* method used that such a blocking mechanism appeared to be present. Although there was no direct relationship between *in vitro* cytotoxic activity and *in vivo* blocking potential using the absorbed sera, it was found that sera with high *in vitro* cytotoxic titres had the greatest blocking potential. However, it was not possible to extend HeLa tumour survival much beyond Day 14 in normal mice treated with absorbed sera before the inoculation of HeLa cells. Histological examination of the nodule so derived showed it to be a HeLa tumour and not an inflammatory response to the inoculation of foreign cells. Normal sera treated with HeLa cells *in vitro* had no blocking effect. These observations are currently being investigated further.

### DISCUSSION

These studies have shown that mice treated with ALS before the inoculation of HeLa cells have a long-term tumour survival rate of 67%. It is generally believed that ALS selectively depresses the cellular immune response (Levey and Medawar, 1966*b*) and that allograft rejection is controlled by this cellular response (Mitchison, 1955). Although our system is not an allograft system, it is reasonable to correlate the graft survivals with depression of this response, which we observed as a result of ALS treatment as well. In untreated mice, tumours will not grow much beyond Day 4.

Medawar (1969) has shown that ALS treated mice do not recover full immunological competence until some 50 days after the end of ALS treatment. In our investigations this would be at about Day 66, ALS treatment having ended on Day 16. However, we have found that the highest rejection rate occurs between Day 33 and Day 60. This observation would appear to contradict Medawar's findings, particularly as graft rejection is controlled by the cellular immune response, unless there is an additional process controlling xenograft rejection. We have found that the high rejection rate of HeLa tumours between Day 33 and Day 60 occurs concurrently with a considerable elevation of the humoral response. Although it may be argued that *in vitro* cytotoxic activity is not necessarily an indication of the *in vivo* state of the immune mechanism, the presence of a high *in vitro* cytotoxic activity during this period of maximal tumour regression seems to be significant, and may be a controlling factor in this system. Raised humoral antibody levels have been reported before in response to tumour xenografts (Beverly and Simpson, 1970).

Our investigations into the presence of a blocking antibody have shown that sera with high cytotoxic titres, measured *in vitro*, appear to have *in vivo* blocking activity. Although we have only succeeded in increasing the HeLa cell tumour mean survival rate from 4 to 14 days, when absorbed sera are passively passaged into normal mice before the inoculation of HeLa cells, we feel that the constancy of the observation must be due to some factor in the sera which is not present in normal sera. The detection of a blocking antibody reinforces the hypothesis that enhancement may be an additional factor contributing to long-term tumour survival, though in view of this limited extension of graft growth using passive serum transfer, its significance is equivocal. Enhancement has been demonstrated many times for allogeneic tumours (Takasugi and Hildemann, 1969*a*, *b*) and recently for syngeneic tumours (Hellström and Hellström, 1970).

The observations reported here do not support the hypothesis that immunological tolerance is a satisfactory explanation for this phenomenon. They fail also to support the hypothesis that long-term survival of tumours is a function of a change in surface antigenicity during growth *in vivo*. Passaged tumours were rejected in untreated mice though the mean time for rejection was increased. This was probably due to the greater size of the passaged HeLa cell implant over and above that given by an initial inoculation of cell suspension.

The tumours that survive are well localized at the site of inoculation, have a good blood supply and are coated with a hypofibrous layer. There is a complete absence of macroscopic and microscopic metastases and the host mice have been shown to reject another human tumorigenic cell line and, furthermore, they rapidly destroy additional inoculations of the original HeLa cells even though they continue to maintain the original HeLa cell tumour. This phenomenon of concomitant immunity has been observed by many investigators in the field of tumour transplantation (Ehrlich, 1906; 1908; Gershon, Carter and Kondo Kazunari, 1967; Carter, 1970), and may be explained by the fact that tumour survival is a result of a local reaction at the graft site.

We feel that long-term tumour survival is not yet fully understood and there may be more than one controlling factor involved. In our investigations we have referred to four possibilities: depression of the cellular response, a local reaction at the graft site, the effect of blocking factor and the elevation of the humoral response. It is suggested that only when these are acting in the same direction tumour survival occurs. It is not surprising therefore that there is a 33% failure rate.

We wish to thank Dr W. D. Brighton of this Division for his advice and considerable help with the immunofluorescence, Dr Missen and Dr Rose (Guy's Hospital) for the histological and haematological analysis, Dr M. Raff, NIMR, Mill Hill for supplying the anti-$\theta$ serum, and Miss D. Reeson for her very competent technical assistance.

### REFERENCES

BEVERLY, P. C. L. & SIMPSON, E. (1970) Humeral Responses to Tumour Xenografts in ALS Treated Mice. *Int. J. Cancer*, **6**, 415.

CARTER, R. L. (1970) Facilitation of Metastatic Growth by Antilymphocyte Serum. *Nature, Lond.*, **226**, 368.

COHEN, A. & SCHLESINGER, M. (1970) Absorption of Guinea-pig Serum with Agar. *Transplantation*, **10**, No. 1, 130.

EHRLICH, P. (1906) Arbeiten aus dem kgl. *Inst. exp. Therap., Frankfurt*, **1**, 65.

EHRLICH, P. (1908) *Verh. dt. path. Ges.*, **12**, 13.

GERSHON, R. K., CARTER, R. L. & KONDO, KAZUNARI. (1967) On Concomitant Immunity in Tumour-bearing Hamsters. *Nature, Lond.*, **213**, 674.

HELLSTRÖM, I. & HELLSTRÖM, K. E. (1970) Colony Inhibition Studies on Blocking and Non-blocking Serum Effects on Cellular Immunity to Moloney Sarcomas. *Int. J. Cancer*, **5**, 195.

LEVEY, R. H. & MEDAWAR, P. B. (1966*a*) Nature and Mode of Action of Antilymphocytic Antiserum. *Proc. natn. Acad. Sci., U.S.A.*, **56**, 1130.

LEVEY, R. H. & MEDAWAR, P. B. (1966*b*) Some Experiments on the Action of Antilymphoid Sera. *Ann. N.Y. Acad. Sci., U.S.A.*, **129**, 164.

MEDAWAR, P. B. (1969) Antilymphocytic Serum. Its Properties and Potential. *Hosp. Pract.*, **4**, 26.

MITCHISON, N. A. (1955) Studies on the Immunological Response to Foreign Tumour Transplants in the Mouse. 1. The Role of Lymph Node Cells in Conferring Immunity by Adoptive Transfer. *J. exp. Med.*, **102**, 157.

PHILIPS, B. & GAZET J. C. (1967) The Growth of Two Human Cell Llines in Mice Treated c̄ Antilymphocyte Serum. *Nature, Lond.*, **215**, 548.

PHILIPS, B. & GAZET, J. C. (1968) Effect of ALS on the Growth of Hep 2 and HeLa Cells in Mice. *Nature, Lond.*, **220**, 1140.

RAFF, M. C. (1969) Isoantigen as a Marker of Thymus-derived Lymphocytes in Mice. *Nature, Lond.*, **224**, 378.

STANBRIDGE, E. J. & PERKINS, F. T. (1969) Tumour Nodule Formation as an *in vivo* Measure of the Suppression of Cellular Immune Response by ALS. *Nature, Lond.*, **221**, 80.

TAKASUGI, M. & HILDEMANN, W. H. (1969*a*) Regulation of Immunity Toward Allogeneic Tumours in Mice. I. Effect of Antiserum Fractions on Tumour Growth. *J. natn. Cancer Inst.*, **43**, 843.

TAKASUGI, M. & HILDEMANN, W. H. (1969*b*) Regulation of Immunity Toward Allogeneic Tumours in Mice. II. Effect of Antiserum and Antiserum Fractions on Cellular and Humoral Response. *J. natn. Cancer Inst.*, **43**, 857.